# Supplementary material for: Domain Wall Automotion in Three-Dimensional Magnetic Helical Interconnectors
Source: ACS Nano. 2022 May 17;16(6):8860–8. doi: 10.1021/acsnano.1c10345 (PMC9245342; doi:10.1021/acsnano.1c10345)
Supplement: Supplementary file 1 — nn1c10345_si_001.pdf [file nn1c10345_si_001.pdf]

Domain wall automotion in three-dimensional magnetic helical interconnectors

Luka Skoric[1,*], Claire Donnelly[1,2], Aurelio Hierro-Rodriguez[3,4], Miguel A. Cascales Sandoval[3], Sandra Ruiz-Gómez[2,5], Michael Foerster[5], Miguel A. Niño[5], Rachid Belkhou[6], Claas Abert[7,8], Dieter Suess[7,8], and Amalio Fernández-Pacheco[9, *]

[1]Department of Physics, Cavendish Laboratory, University of Cambridge, JJ Thomson Ave, Cambridge CB3 0HE, UK
[2]Max Planck Institute for Chemical Physics of Solids, 01187 Dresden, Germany
[3]SUPA, School of Physics and Astronomy, University of Glasgow, Glasgow G12 8QQ, UK
[4]Depto. Física, Universidad de Oviedo, 33007 Oviedo, Spain
[5]ALBA Synchrotron Light Facility, 08290 Cerdanyola del Vallès, Spain
[6]SOLEIL Synchrotron, L'ormes des Merisiers, Saint Aubin BP-48, 91192 Gif-Sur-Yvette Cedex, France
[7]Faculty of Physics, University of Vienna, 1010 Vienna, Austria
[8]Research Platform MMM Mathematics-Magnetism-Materials, University of Vienna, 1010 Vienna, Austria
[9]Insituto de Nanociencia y Materiales de Aragón (INMA). CSIC-Universidad de Zaragoza, 50009 Zaragoza, Spain
[*]E-mail: ls604@cam.ac.uk; amaliofp@unizar.es

S1 X-ray induced bending

We observe a mild deformation of the fabricated structure (Fig. S1a) following the shadow-XPEEM measurements. This is likely the result of the invasive shadow-XPEEM measurements that expose the structure to high voltages, and extreme X-ray radiation with only narrow heat sink at the base of the structure.[1] Also, the high heat and vacuum conditions could lead to the release of gases trapped in the scaffold's and graphitization of carbonaceous Pt matrix,[2,3] leading to the structural changes of the scaffold.[4] As the shape of the structures' PEEM shadow is the same at the beginning and end of the measurements (Fig. S1b), this is expected to have happened upon the initial exposure to the PEEM environment, after which the structure was stable.

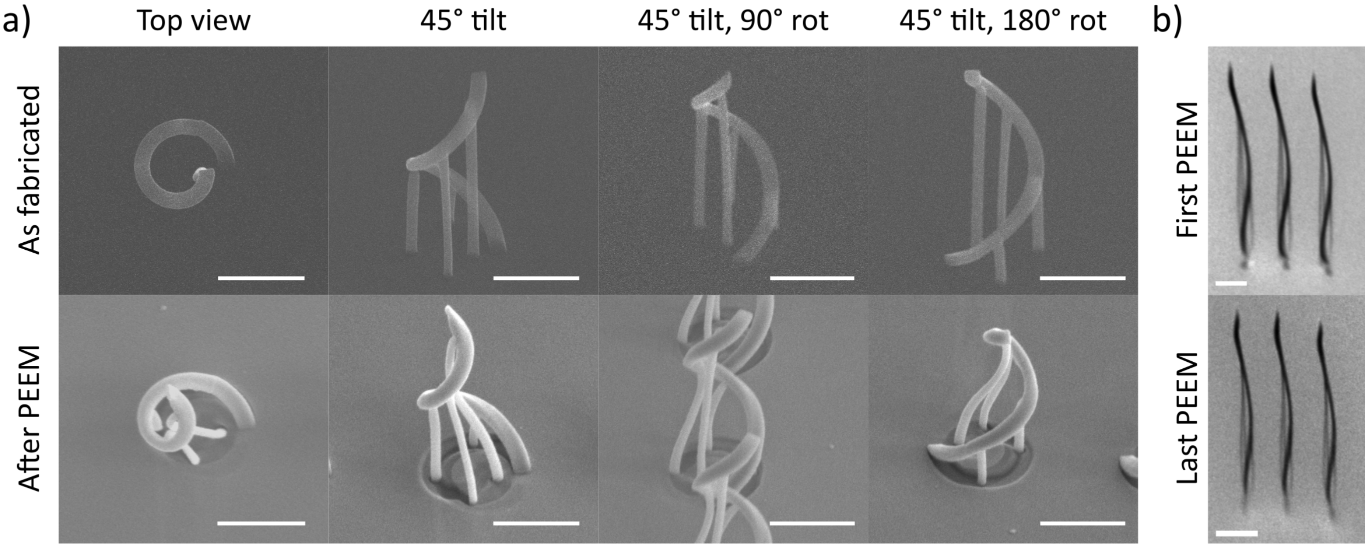

Figure S1: Fabricated structure before and after PEEM. (a) Scanning Electron Microscope (SEM) images comparing the FEBID-fabricated structure (top row) with the same structure after PEEM measurements (bottom row). The angle of viewing is specified above each column. (b) First and last PEEM images of the structures shadow showing no noticeable change in the geometry. All scale bars are 1 μm.

S2 Simulation structure details

A 3D model of the fabricated structure closely matching the structure after PEEM experiments was created (Fig. S2a-c). The model was designed with Autodesk Inventor to be a $h = 2.6$ µm tall, $n_{rev} = 1.2$ revolutions (440°) spiral with the initial radius $R_0 = 1.15$ µm, and $\beta = -9°$ taper. The equation of the spiral central line in cylindrical coordinates is:

$$\begin{aligned} r(z) &= R_0 + z\tan(\beta) \\ \varphi(z) &= 2\pi n_{rev}\frac{z}{h}. \end{aligned} \tag{S1}$$

Since the magnetic film has been deposited using unidirectional thermal evaporation and the spiral shape was unchanged by this process (see Sec. S1), we expect the film to be of a uniform thickness in the direction perpendicular to the substrate (*i.e.* parallel to the spiral axis). Therefore, to construct the model with thickness gradient matching the deposited film (see Fig. 3 in the main text), a 50 nm thick, 150 nm wide profile is swept parallel to the spiral axis. Parametrically, the resulting magnetic volume is described by:

$$\begin{aligned} r(\zeta,\omega,\tau) &= R_0 + \zeta\tan(\beta) + \omega \\ \varphi(\zeta,\omega,\tau) &= 2\pi n_{rev}\frac{\zeta}{h} \\ z(\zeta,\omega,\tau) &= \zeta + \tau \\ \zeta \in [0, 2600\text{ nm}];\ \tau &\in [-50\text{ nm}, 0];\ \omega \in [-75\text{ nm}, 75\text{ nm}] \end{aligned} \tag{S2}$$

where ζ, ω and τ are parameters specifying spiral height, width and film thickness respectively.

In order to achieve a uniform thickness for comparison, another model with the same profile swept normal to the spiral curve was constructed. The model was tilted by 8.7° to z axis in the plane 45° to the yz plane to match the fabricated spiral. Based on the central line formula (Equation S1), the tangent $\mathbf{t}$ and the curvature κ of the spiral can be calculated. The thickness of the film normal to the spiral is given by $T_n = 50\sqrt{1-t_z^2}$ nm where t_z is the component of the tangent in the z direction.

We plot both the film thickness and the curvature as functions of the arc length s along the spiral curve (Fig. S2d,e). We find that the average thickness gradient is -5.3 nm/µm. The curvature gradient in the center of the spiral ($s = 1750$ nm), where the domain wall (DW) is initialized in simulations used for Fig. 3 in the main text, is 0.09 µm^{-2}.

Using the results of the previous theoretical works on the curvature-induced DW automotion,[5] an approximation of the expected DW speed due to the gradient in curvature can be acquired. According to Ref. 5 asymptotic expression for the magnitude of DW velocity is

$$V = \pi\Delta_0\gamma_0 M_s\frac{\chi\ell^2}{\alpha} \tag{S3}$$

where χ is the curvature gradient, ℓ the exchange length, α the damping constant, γ_0 is the gyromagnetic ratio, M_s the saturation magnetization, and $\Delta_0 = \ell/\sqrt{k_a}$ is the DW width with k_a being the dimensionless effective shape anisotropy depending on the ratio of width and thickness $\delta = w/T_n$ as:[5]

$$k_a = \frac{1}{2\pi}\left(\frac{1-\delta^2}{2\delta}\ln(1+\delta^2) + \delta\ln\delta + 2\arctan\frac{1}{\delta}\right) \tag{S4}$$

We set $w = 150$ nm and $T_n = 40$ nm (see Figure S2) for the width and thickness of the magnetic conduit. Using $\chi = 0.09$ µm^{-2}, $\alpha = 0.01$, $M_S = 8\times10^5$ Am^{-1}, $A = 1.3\times10^{-11}$ Jm^{-1}, and $\gamma_0 = 2.2128\times10^5$ m/As, we obtain $V \approx 2.5$ ms^{-1}. This expected low velocity explains the comparatively low contribution of the curvature-induced automotion to the overall effect.

However, it is important to note that the theoretical works have been focusing on describing the motion of transverse DWs,[5] while in our systems, we expect to have vortex DWs, which may affect the accuracy of the estimation. We hope that this work will stimulate further theoretical exploration of automotion in nanowires with different DW configurations.

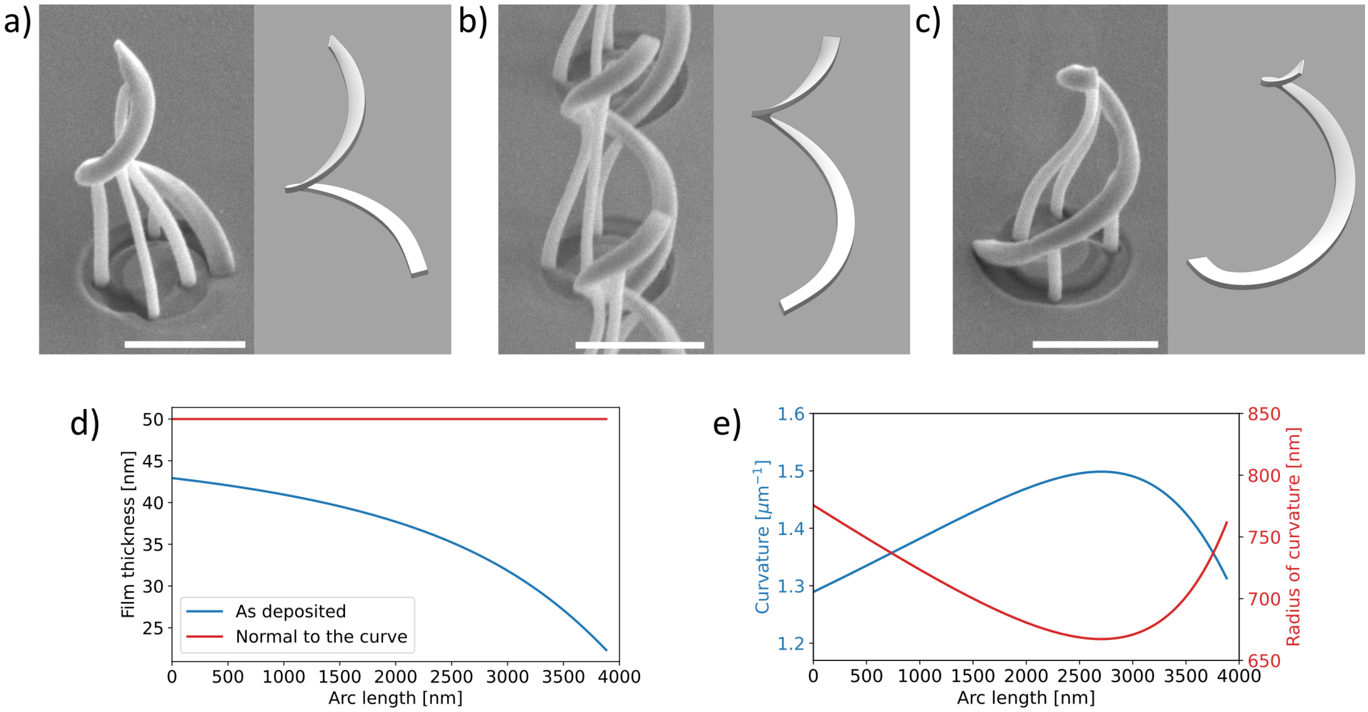

Figure S2: Simulation model details. (a-c) Comparison of the SEM images (left) of the fabricated structure after PEEM experiments, with the CAD model used for simulations (right). All views are at 45° tilt. (b) and (c) are respectively rotated by 90° and 180° with respect to (a). (d) Thickness of the film as a function of arc length for the as deposited structure, and the structure with profile swept normal to the spiral curve. (e) Curvature, and the radius of curvature as functions of arc length.

S3 Calculating DW position

In order to track the DW motion in the simulations, its position at each simulation snapshot (Fig. S3a) needs to be extracted. To do this, the structure's stereolithography (STL) file is sliced into 1000 slices of equal height increments, not unlike slices in 3D printing. At each slice, the centroid of the bounding polygon is found, giving a sequence of points representing the central spiral line (blue dots in Fig. S3b). Arc length along the spiral is acquired by integrating along the path connecting the central points. Furthermore, the tangent to the spiral at each slice is given by the unit vector pointing from the center of the current slice to the next one. To acquire the DW profile, the average magnetization in each slice is projected onto the tangent (see data in Fig. S3c). Within a single domain the magnitude of the projection is close to 1, with the transition between the domains given by the DW. The data is fitted with the Walker trial function:[6,7]

$$
\begin{aligned}
(\mathbf{m}\cdot\mathbf{t})(x) &= \cos\theta(x) \\
\theta(x) &= 2\arctan\left(\exp\left(\frac{x-x_0}{\Delta}\right)\right)
\end{aligned}
\tag{S5}
$$

where Δ, and x_0 are the fitting parameters (orange line in Fig. S3c). The DW width δ_W is determined by Δ through $\delta_W = \pi\Delta \approx 330$ nm,[7] while the location of the DW is given by x_0. The resulting location (red line in Fig. S3c) is used in the subsequent analysis of the domain dynamics in the main text.

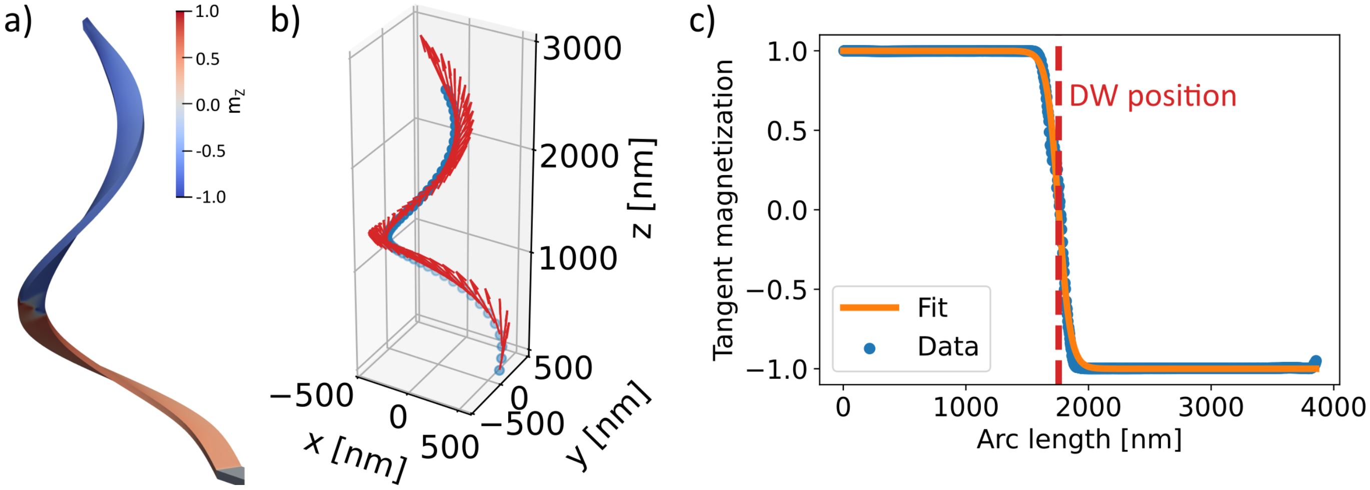

Figure S3: Calculating DW position from the simulation results. (a) A snapshot of the simulation with the DW about half-way along the structure. (b) The central points of the structure slices (blue dots), and the tangent to the structure (red arrows). (c) The projection of the average magnetization in each slice onto the tangent to the spiral (blue dots), and the fit to the DW profile (orange line). The calculated DW position is marked with the red dashed line.

S4 Domain wall speed

From the simulations in the Fig. 3 in the main text, we can deduce the evolution of the DW speed during the motion. For the structure where the motion is driven by the edge effects only, we observe a largely constant low velocity of about 30 m/s for the majority of motion (Fig. S4a). As the DW comes to the vicinity of the top (for $t > 50$ ns), it experiences an increasingly stronger pull from the magnetic charges at the edge of the structure, leading to a strong acceleration and the subsequent annihilation.

When the motion is driven by thickness gradient as the ones present in our structures, we observe a significantly faster motion. During the first 10 ns, the DW accelerates reaching the speed of over 200 m/s. The exceedingly fast motion results in the Walker breakdown where the precessional motion of the DW is observed. As the thickness gradient is the dominant effect, combining it with the edge effects introduces only a small increase in the driving force. In this case, the initial acceleration is even faster, and the Walker breakdown is reached about 1 ns earlier.

It is worth noting that the theoretically predicted value for the Walker breakdown speed agrees with the one obtained here. In particular, prior to the Walker breakdown, the domain wall velocity can be approximated by:

$$v = \frac{\gamma_0 \Delta}{\alpha} H \tag{S6}$$

where γ_0 is the gyromagnetic ratio, $\Delta = \sqrt{A/K_u}$ is the DW width parameter and α is the Gilbert damping parameter.[8,9] Using $H_W = \alpha H_K/2$ where H_K is the demagnetizing field, and the simulation parameters as defined in the main text, the speed just prior to the Walker breakdown becomes:

$$v_W = \frac{1}{2}\gamma_0 \Delta H_K \approx 250 \text{ m/s}, \tag{S7}$$

agreeing with the numerical simulations and previous similar numerical studies[10] (Fig. S4b).

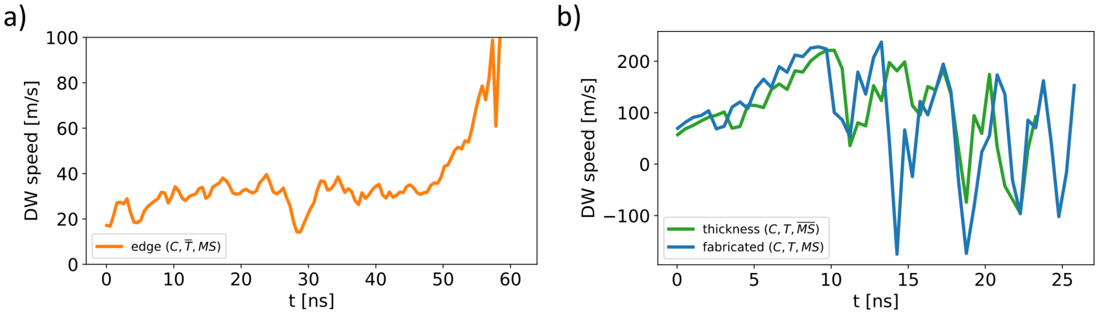

Figure S4: Speed of the DW motion as a function of time. (a) The motion driven by the edge effects. (b) The motion driven by the thickness gradient (green), and the combination of the thickness gradient and the edge magnetostatics in the as-fabricated model (blue). After about 10 ns as the DW speed exceeds 200 m/s, we observe the Walker breakdown characterized by the back-and-forth oscillatory motion. See Fig. 2 in the main text for the definition of models.

S5 Walker breakdown

As the speed of the DW exceeds 200 m/s, it enters into a Walker breakdown regime, characterized by the precessional motion and periodic internal transformations of the DW spin structure.[6] In Fig. S5, we show the snapshots during such transformation where the vortex wall core (Fig. S5, part 1) is pushed to the side of the wire (Fig. S5, part 2), flipping the polarity (Fig. S5, parts 3 and 4). This is accompanied by halting of DW motion, followed by the reverse displacement. The energy stored in the DW during the motion[11] is released in the form of spin waves (Fig. S5, part 1, 2). These typical features characteristic of the Walker breakdown under field and current driven motion are thus also observed under DW automotion for the spiral interconnectors under investigation, when high DW speeds are reached.

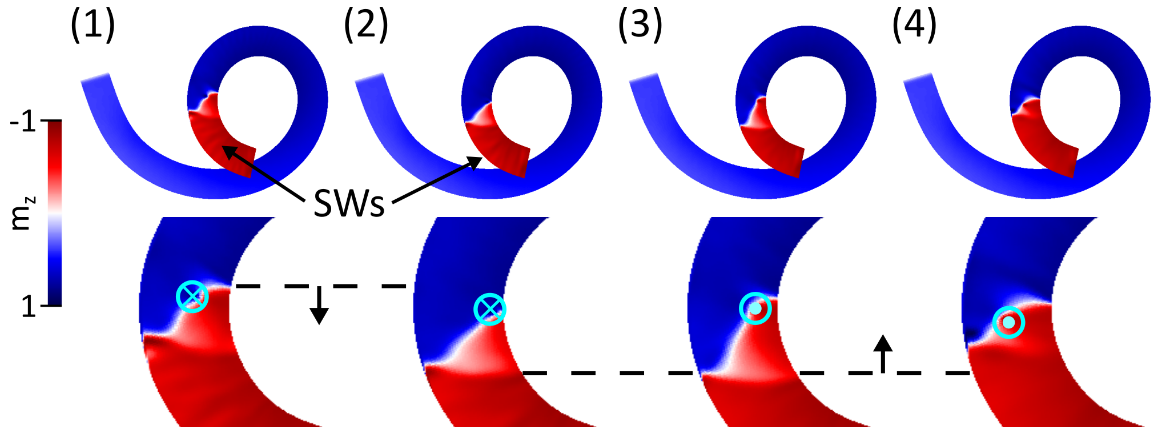

Figure S5: Dynamics beyond the Walker breakdown. The top view of the simulated structure is shown colored by the z component of magnetization (top row) together with the focused view on the DW region (bottom row). As the DW speed goes over 200 m/s it experiences the Walker breakdown. (1-2) the core of the vortex wall is pushed to the side of the spiral where (2-3) it flips the polarity and (3-4) reforms the vortex wall of the opposite polarity while in the opposite direction (denoted by the arrow). During the process, spin waves (SWs) are emitted, most apparent as ripples in (1) and (2).

S6 Mesh size

In order to ensure that the mesh size does not affect our results, we compare the DW automotion simulations of the fabricated structure at two different mesh sizes: 5.7 nm as used in all simulations in the main paper, and 4 nm (see Fig. S6). Overall, the two plots are equivalent with initial DW accelerations matching almost perfectly and only a small difference during the Walker breakdown oscillations.

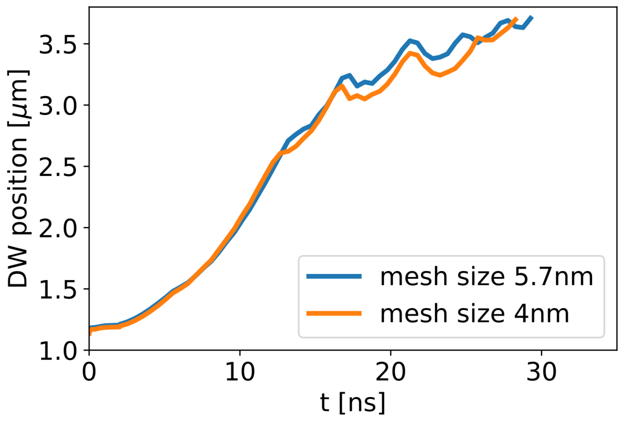

Figure S6: Mesh size comparison. Simulations of DW automotion for mesh sizes of 5.7 nm and 4 nm.

S7 XAS spectra

Prior to X-ray magnetic circular dichroism (XMCD) measurements, the X-ray absorption spectroscopy (XAS) was measured on the structure's shadow to optimize the energy for maximum magnetic contrast (Fig. S7a). The XAS data was acquired by taking shadow- Photo Emission Electron Microscopy (PEEM) images at different energies. Following the normalization and alignment of images, the average of the signal in a selected region on the structure shadow is taken for each energy (see the red area in Fig. S7b). The signal is normalized by the average of the off-edge data ($E > 730$ eV). The resulting data points are fitted with a double-Gaussian function:

$$f(E; A, C_1, C_2, \mu_1, \mu_2, \sigma_2) = A + C_1 \exp^{-\frac{(E-\mu_1)^2}{2\sigma_1^2}} + C_2 \exp^{-\frac{(E-\mu_2)^2}{2\sigma_2^2}} \tag{S8}$$

where E is the beam energy, and all the other parameters are fitting parameters (see fits in Fig. S7a).

We find Fe L_3 edge at 708.5 eV, and L_2 edge at 722 eV, which is in good agreement with XAS spectra on $Ni_{80}Fe_{20}$ films.[12,13] The absence of shoulders or double peaks in the spectrum (Fig. S7a) confirms that the capping layers worked well, and the magnetic film is not oxidized. Furthermore, the expected opposite sign of the normalized intensity difference between the two edges is observed, with L_3 having the larger magnitude (bottom of Fig. S7a). In order to verify that the contrast in the shadow is indeed of magnetic character, shadow-XPEEM images are taken at both $L_{3,2}$ edges (Fig. S7b,c). As expected, the image taken at L_3 (Fig. S7b) has an inverse contrast to L_2 (Fig. S7c). The L_3 edge gives a stronger XMCD, resulting in the better magnetic contrast. Therefore, the imaging energy was set to L_3 edge (708.5 eV) for all further experiments.

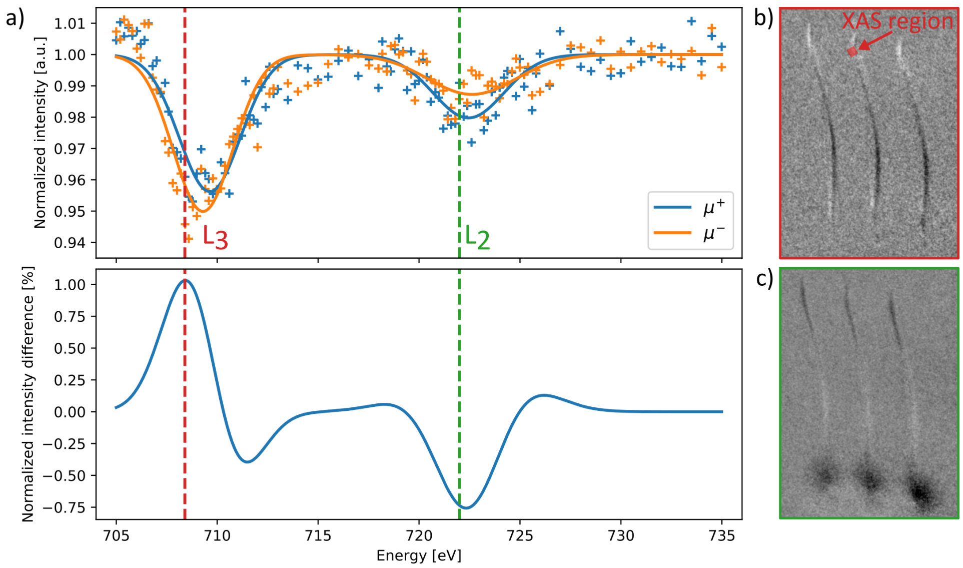

Figure S7: XAS spectrum of the structure shadow. (a) Top: Fe $L_{3,2}$-edge XAS with double-Gaussian fits for the normalized signals for two polarizations μ^+ and μ^-. Bottom:the difference of the fits. Red and green dashed lines denote the energies that were used to measure at L_3 (708.4 eV), and L_2 (722 eV) edges respectively. Images taken at (a) L_3, and (b) L_2 edges showing the opposite contrast. The region taken for XAS is depicted by the red square on (b).

S8 Shadow-XPEEM image processing

In shadow-XPEEM, the measured intensity of photoelectrons released by the top layer of the substrate is proportional to the transmitted ray intensity. The transmitted intensity I_1 decays exponentially with the thickness of the material d that the ray is going through:

$$I_1(E, d) = I_0(E) \exp^{-\mu(E) \cdot d} \tag{S9}$$

where I_0 is the initial beam intensity, and μ is a material-dependent absorption coefficient.[13] The magnetic contrast in XMCD arises from the dependence of the absorption coefficient on the relative orientation of the X-ray beam $\mathbf{k}$, and the direction of magnetization $\mathbf{m}$:

$$\mu^{\pm} = \mu_0 \pm \delta(\mathbf{m} \cdot \mathbf{k}) \tag{S10}$$

where $\mu^{\pm}$ corresponds to the absorption coefficient of the circularly-right (CR) and -left (CL) polarization, μ_0 is the non-magnetic absorption of the material, and δ is the change in absorption due to XMCD. Therefore, the difference of logs of intensities $I^{\pm}$ of the two circular polarizations is proportional to the XMCD signal:

$$\log\left(\frac{I^+}{I^-}\right) \propto d(\mathbf{m} \cdot \mathbf{k}) \tag{S11}$$

Shadow-XPEEM images are acquired by taking a stack of 128 snapshots using circularly-right (CR) and -left (CL) polarizations (Fig. S8, part 1). Firstly, to remove the detector artifacts, the images are normalized by dividing them by a defocussed image containing no structures (Fig. S8, part 2). The brightness of all images is equalized to adjust for any beam drift during the acquisition. Secondly, the normalized stacks are aligned using phase cross-correlation to get translational offsets between the images.[14] The average of each of the aligned stacks is taken, resulting in two low noise images (Fig. S8, part 3). Finally, the two images are again aligned using phase cross-correlation, and the log of ratio of intensities is taken, yielding the XMCD signal according to Equation S11.

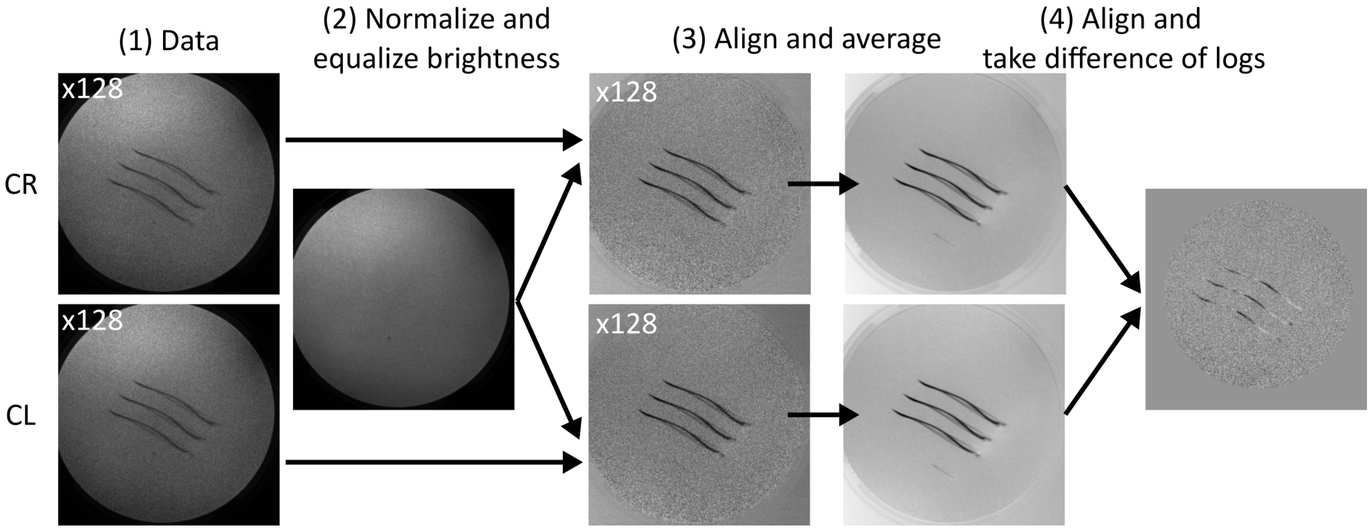

Figure S8: Image processing pipeline. For each of the right (CR) and left (CL) circular polarizations, (1) the acquired stack of 128 images is (2) normalized and the brightness of images equalized. (3) The images within each stack are aligned, and the average is taken. (4) Finally, the two polarizations are put together, aligned and the difference of logs is taken to acquire the XMCD contrast.

S9 Shadow-XPEEM simulations

The results of the experimental states are correlated with the simulations of the shadow-XPEEM based on the magnetic states acquired in micromagnetic simulations. The simulation results consists of an array of tetrahedra defining the mesh, and the corresponding per-vertex magnetization. Firstly, the outline of the structure is projected onto the xy plane, acquiring the structure's shadow. Each point i in the shadow, was used as the base of the ray in the direction of the X-ray beam $\mathbf{k}$, finding which mesh tetrahedra it intersects (Fig. S9a). Further, for each intersected tetrahedra j, the intersection distance d_j, and the average magnetization of tetrahedra vertices $\mathbf{m_j}$ was determined (Fig. S9b). The XMCD signal, as defined is Equation S11, is given by the sum over individual contributions from all the intersected tetrahedra I_i:

$$\text{XMCD}_i \sim \sum_{j \in I_i} d_j \mathbf{k} \cdot \mathbf{m}_j. \tag{S12}$$

The background of the image is set to 0 XMCD ($\text{XMCD}_{\text{back}} = 0$). Finally, the contrast and brightness of the image are adjusted, and the Gaussian blur with $\sigma = 4$ applied to match the experimentally acquired images (Fig. S9c).

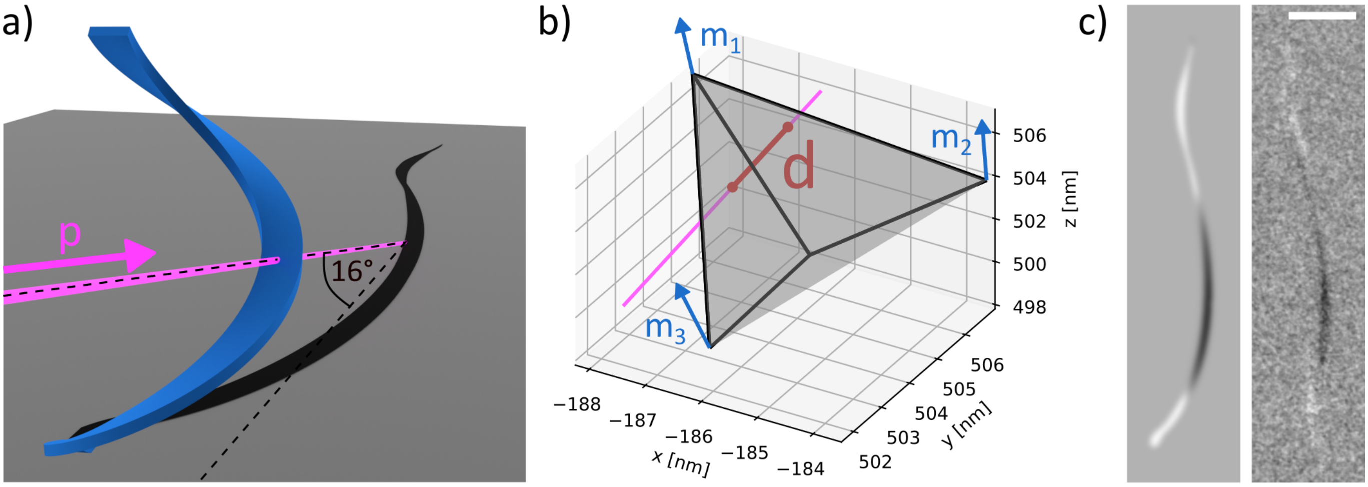

Figure S9: Shadow-XPEEM projection simulations. (a) The projection simulations is acquired by taking each pixel in the structure's shadow, and finding which tetrahedra the ray to that pixel intersects. (b) For each intersected tetrahedron, the intersection distance d and average magnetization $\mathbf{m}$ are found, and the corresponding summand $d\mathbf{k} \cdot \mathbf{m}$ is calculated. (c) Summing over the intersected tetrahedra for each of the shadow points, the simulated shadow-XPEEM contrast (left) is determined, being subsequently compared with the measured structure (right). In this example, a good matching between both images is found, corresponding to a single domain state. The scale bar is 1 µm.

S10 Domain wall height

In order to measure the DW position along the shadow length (as in Fig. 3 and Extended Data Fig. 1 in main text), we combine the data from the structure shadow with the XMCD image. Firstly, the image of the shadow is acquired by averaging the PEEM images using CL and CR polarizations (Fig. S10a, part i). The shadow image is thresholded and only the XMCD signal under the shadow of individual structures is taken (Fig. S10a, part ii and iii). The signal is averaged in slices perpendicular to the X-ray direction to acquire the signal as a function of the position along the shadow (Fig. S10c). The same procedure has also been applied to the simulations (Fig. S10b and bottom graph in Fig. S10c). While the single-doman states have a smoothly oscillating contrast (orange lines in Fig. S10c), the DW is signified by the quickly changing bright-dark region (green circles in Fig. S10c). Since the beam is incident at 16° glancing angle, the height H of the DW on the structure can be related to its position P along the shadow via $H = P\sin(16°)$. Thus, this measurement allows us to correlate the position of the DW on the shadow with the location on the structure.

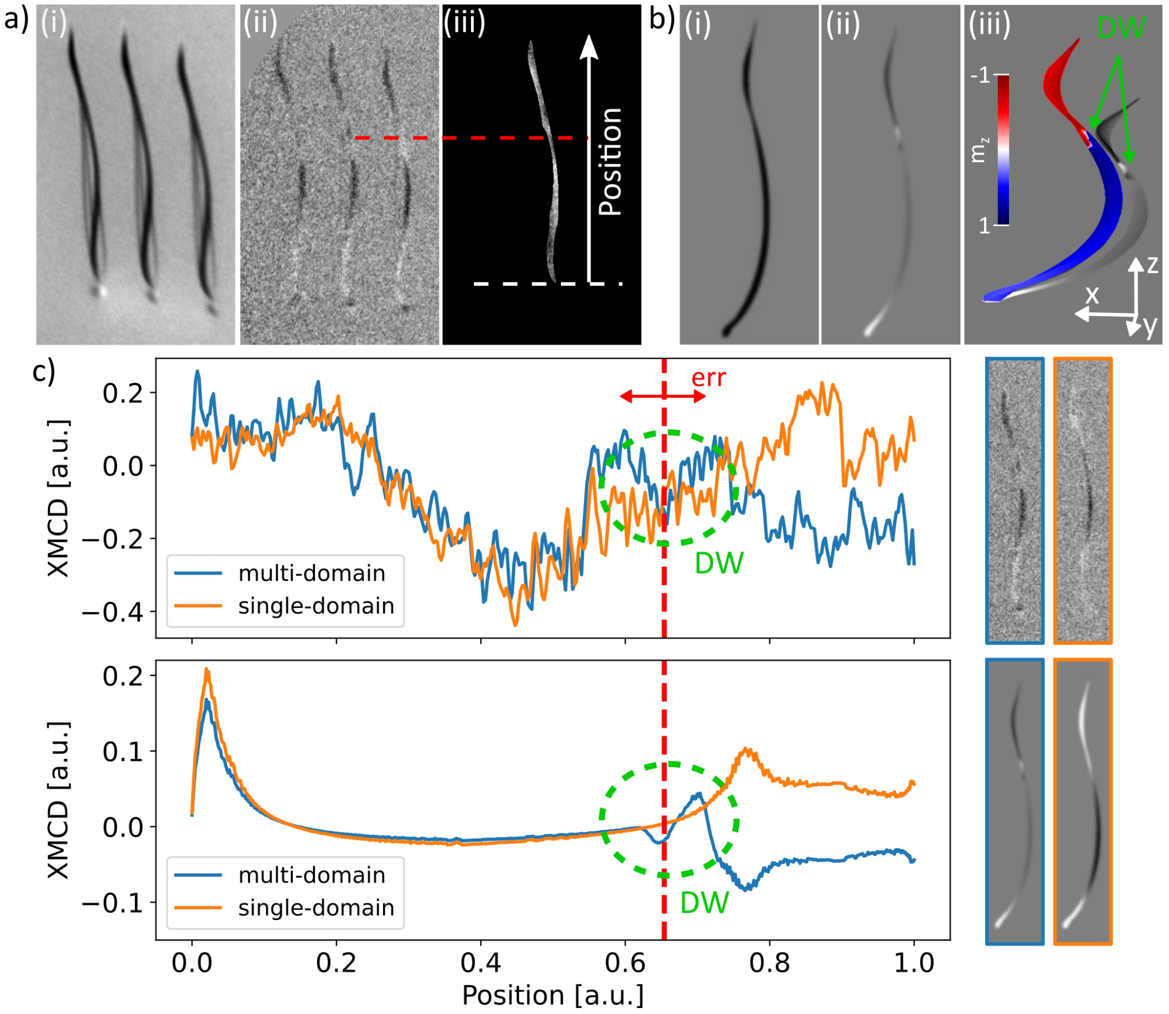

Figure S10: Measuring DW position. The relative height of the DW is measured by finding its position on the shadow with respect to the total shadow length. (a) The average image from the CL and CR polarizations (i) is used to extract only the XMCD signal (ii) in the shadow. (iii) Individual structures are selected and the XMCD signal plotted as a function of the position on the shadow. The location of the DW is marked with red dashed line. (b) For comparison, same can be done on the simulated shadow (i) and the corresponding XMCD signal (ii). The side view of both the structure (colored) and the shadow is shown in (iii). It can be seen how the location of the DW corresponds to the rapidly changing contrast in the shadow (green arrows). (c) Normalized XMCD signal is plotted as a function of position along the shadow for the measured data (top) and the simulations (bottom). Blue lines in the top and bottom axes show the data from the multi-domain state in (a) and (b) respectively. In both cases, it is compared to the corresponding single-domain states plotted in orange. The location of the DW is shown by the red dashed line and also corresponds to the location extracted directly from the simulations using the method in Section S3.

S11 Domain wall chirality

In simulations, the circulation of the initialized vortex wall depends on the direction and tilt of the magnetic field. For instance, in the "initialize and release" experiment (Fig. 4 in the main text), both right and left circulations can be initialized by introducing a small (10°) tilt in the magnetization direction below or above the horizontal plane respectively. Moreover, the Walker breakdown induced transformation are also known to change the chirality of the DW.[15] With opposite VW circulations having a slightly different contrast pattern, this can affect the determination of the exact DW position (Fig. S11). However, the induced uncertainty is not larger than the DW width $\delta_W \approx 330$ nm (Section S3). The procedure followed in Section S10 is thus a robust way to determine the DW position from the shadow, and allows us to assess in detail the automotion effect in our structures under different protocols, as explained in the main text.

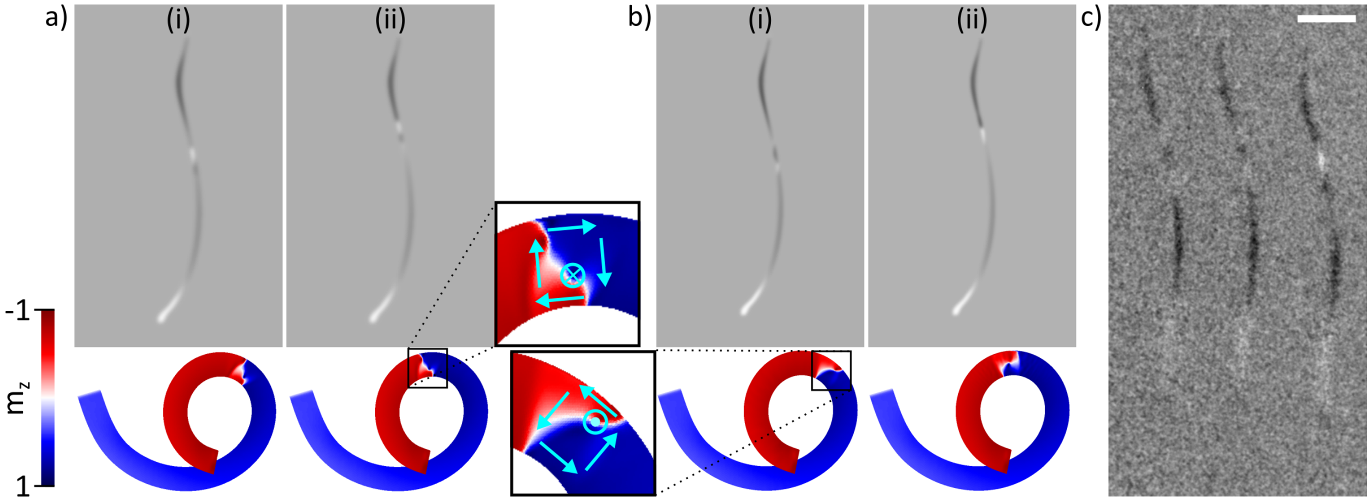

Figure S11: Comparison of DWs with left (a) and right (b) circulation. For both walls, snapshots of two positions are shown with the corresponding shadow-XPEEM simulations on the top. Insets show a closer look at the DW configuration with magnetization direction shown in cyan. Both (a-ii) and (b-i) have a contrast matching the experimentally measured state in (c), despite having slightly different DW positions.

S12 Full angular study

To demonstrate the consistency of the DW motion, an array of three identical structures was measured (Fig. S12). For clarity, in the main text we include the data from only the middle structures, but the equivalent behavior was observed across the whole array. This confirms that the DW automotion in our structures is a robust and reproducible mechanism for DW transport.

We note several features not discussed in the main text in the interest of brevity. Firstly, in some images (specifically 105°, 135°, 180°, and 240°), additional contrast can be seen at the bottom of the shadows. This corresponds to the direct images of the 3D structures. The contrast here usually arises due to the distortion caused by charging of 3D structures in high voltage fields present during XPEEM experiments.

Furthermore, several cases where more than one DW is present can be observed (e.g. 255° right spiral, 285° left spiral, 270° middle spiral). We interpret this to be a state where the top DW is pinned and the bottom one approached it, but did not fully annihilate due to several possible factors, as described in the main text.[15,16]

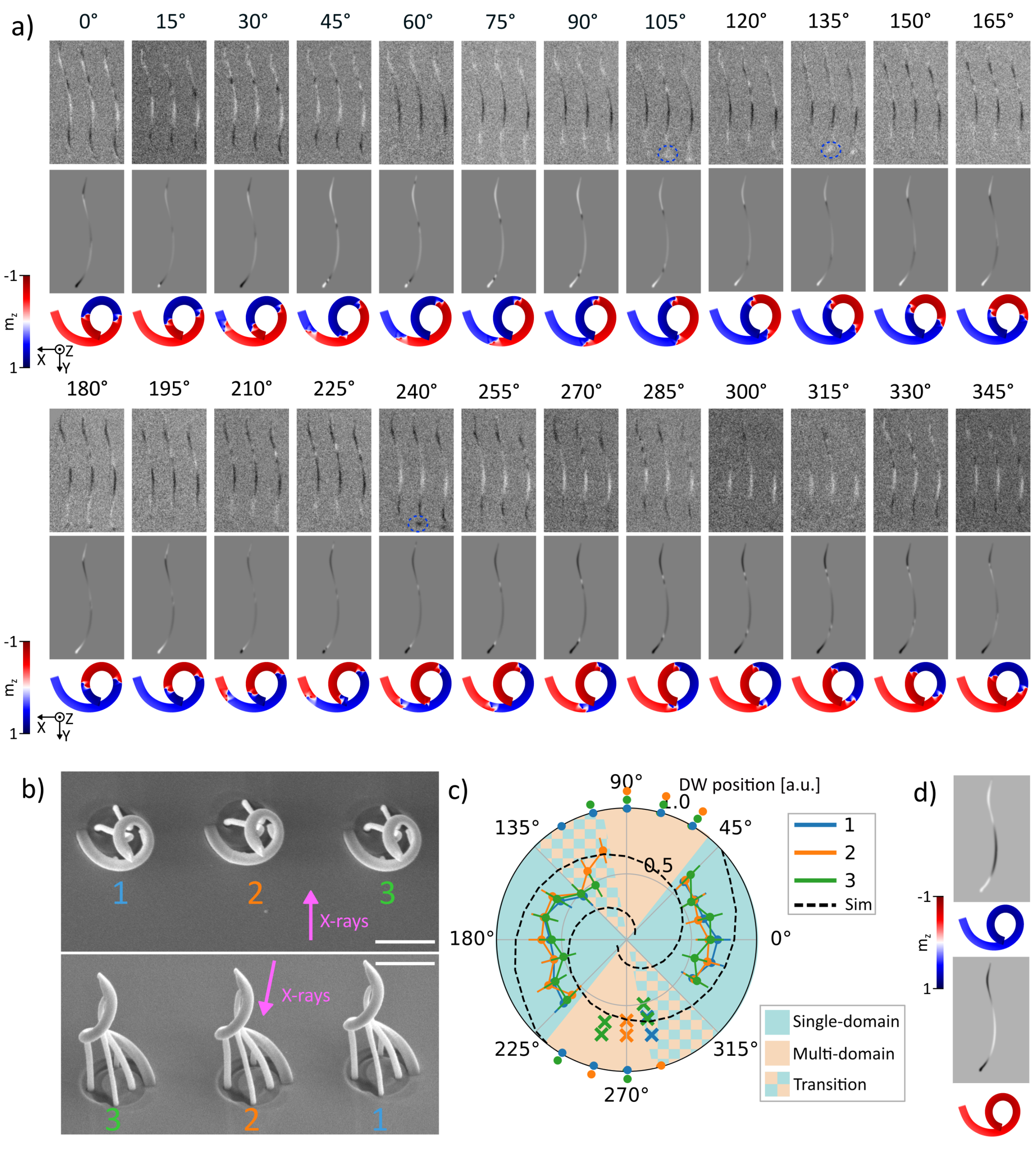

Figure S12: Angles study. (a) The shadow-PEEM images of the system are shown after initialization at different angles with 15° increments. Below each image are the shadow of the simulated state immediately after the initialization (before automotion), and the top view of the simulated state colored by the z component of magnetization. All observed states can be acquired by translating the simulated DWs upwards through automotion (see main text for details). The only exception are the states at 30° and 45° and their inverses at 210° and 225° where in simulations, a formation of a small third domain at the bottom of the spiral is observed. This is likely due to a small disagreement between the fabricated model and the simulated structure near the connection with the substrate. Blue circles (105°, 135°, 240°) correspond to the contrast due to the distortion caused by some charging produced by the 3D structures. (b) SEM images of the array of the three measured spirals: top view (top), and 45° tilt view (bottom). Scale bars are 1 µm. The direction of X-rays is shown in pink. (c) Polar plot of the DW position extending the plot in Fig. 3b in the main text. The measured data for all three spirals is shown. In the main text, only results from the spiral 2 are shown. (d) Simulated singe-domain images for reference.

References

[1] A. Wartelle, J. Pablo-Navarro, M. Staňo, S. Bochmann, S. Pairis, M. Rioult, C. Thirion, R. Belkhou, J. M. de Teresa, C. Magén, and O. Fruchart. Transmission XMCD-PEEM imaging of an engineered vertical FEBID cobalt nanowire with a domain wall. *Nanotechnology*, 29(4):045704, December 2017.

[2] Dhaval D. Kulkarni, Konrad Rykaczewski, Srikanth Singamaneni, Songkil Kim, Andrei G. Fedorov, and Vladimir V. Tsukruk. Thermally Induced Transformations of Amorphous Carbon Nanostructures Fabricated by Electron Beam Induced Deposition. *ACS Appl. Mater. Interfaces*, 3(3):710–720, March 2011.

[3] Harald Plank, Thomas Haber, Christian Gspan, Gerald Kothleitner, and Ferdinand Hofer. Chemical tuning of PtC nanostructures fabricated via focused electron beam induced deposition. *Nanotechnology*, 24(17):175305, 2013.

[4] Ivo Utke, Johann Michler, Robert Winkler, and Harald Plank. Mechanical Properties of 3D Nanostructures Obtained by Focused Electron/Ion Beam-Induced Deposition: A Review. *Micromachines*, 11(4):397, April 2020.

[5] Kostiantyn V. Yershov, Volodymyr P. Kravchuk, Denis D. Sheka, Oleksandr V. Pylypovskyi, Denys Makarov, and Yuri Gaididei. Geometry-induced motion of magnetic domain walls in curved nanostripes. *Phys. Rev. B*, 98(6):060409, August 2018.

[6] N. L. Schryer and L. R. Walker. The motion of 180° domain walls in uniform dc magnetic fields. *J. Appl. Phys.*, 45(12):5406–5421, December 1974.

[7] Olivier Fruchart. *Nanomagnetism.* Institut Neel (CNRS & Universite Grenoble - Alpes), 2014.

[8] Matthew T. Bryan, Thomas Schrefl, Del Atkinson, and Dan A. Allwood. Magnetic domain wall propagation in nanowires under transverse magnetic fields. *J. Appl. Phys.*, 103(7):073906, April 2008.

[9] A. Mougin, M. Cormier, J. P. Adam, P. J. Metaxas, and J. Ferré. Domain wall mobility, stability and Walker breakdown in magnetic nanowires. *EPL*, 78(5):57007, May 2007.

[10] Jusang Yang, Corneliu Nistor, G. S. D. Beach, and J. L. Erskine. Magnetic domain-wall velocity oscillations in permalloy nanowires. *Phys. Rev. B*, 77(1):014413, January 2008.

[11] Jean-Yves Chauleau, Raphaël Weil, André Thiaville, and Jacques Miltat. Magnetic domain walls displacement: Automotion versus spin-transfer torque. *Phys. Rev. B*, 82(21):214414, December 2010.

[12] Xinmao Yin, Weiqiang Sun, Wee Tee Soh, Ping Yang, Xiaojiang Yu, Mark B. H. Breese, Andrew T. S. Wee, Andrivo Rusydi, and Chong Kim Ong. Unraveling the magnetic coupling in the interface of the exchange-biased IrMn/permalloy multilayers. *Materials Letters*, 187:133–135, January 2017.

[13] P. Fischer. X-Ray Imaging of Magnetic Structures. *IEEE Trans. Magn.*, 51(2):1–31, February 2015.

[14] Stéfan van der Walt, Johannes L. Schönberger, Juan Nunez-Iglesias, François Boulogne, Joshua D. Warner, Neil Yager, Emmanuelle Gouillart, and Tony Yu. Scikit-image: Image processing in Python. *PeerJ*, 2:e453, June 2014.

[15] E. R. Lewis, D. Petit, A.-V. Jausovec, L. O'Brien, D. E. Read, H. T. Zeng, and R. P. Cowburn. Measuring Domain Wall Fidelity Lengths Using a Chirality Filter. *Phys. Rev. Lett.*, 102(5):057209, February 2009.

[16] Mohamad-Assaad Mawass, Kornel Richter, Andre Bisig, Robert M. Reeve, Benjamin Krüger, Markus Weigand, Hermann Stoll, Andrea Krone, Florian Kronast, Gisela Schütz, and Mathias Kläui. Switching by Domain-Wall Automotion in Asymmetric Ferromagnetic Rings. *Phys. Rev. Applied*, 7(4):044009, April 2017.
